# Supplementary material for: Are carbon emissions trading and green financial instruments synergistic? -Comprehensive quantitative research based on content analysis
Source: PLoS One. 2024 Mar 7;19(3):e0298601. doi: 10.1371/journal.pone.0298601 (PMC10919601; doi:10.1371/journal.pone.0298601)
Supplement: S1 Data — (ZIP) [file pone.0298601.s001.zip › Data/地级市Z_F工作报告.zip!/╜¡╦╒/─╧═¿╩╨/└┤╫╘╣1⁄2╓┌║┼í╛┬φ┐╦╩2╛▌═°í┐_2010.docx]

各位代表：

　　现在，我代表市人民政府向大会作工作报告，请予审议，并请市政协委员和其他列席人员提出意见。

一、2009年工作回顾

　　过去一年，面对国际金融危机的严重冲击，全市上下在省委、省政府和市委的正确领导下，深入贯彻落实科学发展观，围绕“扎实保增长、全面达小康”工作主线，抢抓长三角一体化发展和江苏沿海开发两大国家战略机遇，落实中央、省的决策部署和政策措施，圆满实现了“保增长”、“达小康”两大目标，较好完成了市十三届人大二次会议确定的各项任务。

　　2009年，实现GDP2872.8亿元，按可比价计算增长14%，增幅继续位居全省前列；财政总收入486.1亿元，其中地方一般预算收入198.99亿元，比上年分别增长24.6%、24.7%；节能减排约束性目标实现；全社会固定资产投资1802亿元，增长19.7%；城镇居民人均可支配收入19468元，农民人均纯收入8696元，分别增长11%和11.3%；城镇登记失业率2.8%。通州、海门、如皋、启东地方一般预算收入均突破20亿元。

　　2009年，省定全面小康25个指标总体达标，在全省江北率先以市为单位达到全面小康标准。

　　（一）积极应对国际金融危机，主要经济指标增幅继续高于全省平均水平。受国际金融危机影响，2009年初，我市不少行业出现负增长，相当数量企业经营困难，面临关停风险。我们坚决贯彻落实上级一系列政策措施，按照市委“危中抓转机、难中攀新高”的要求，正确分析形势，从容应对挑战，加强分类指导，强化要素保障，帮助企业渡过难关，促进经济全面回升。密集出台我市扩大内需促进经济增长的十项措施和优化投资环境等30多个政策文件。跟踪解决项目报批、用地、拆迁难题，连续推动工业、服务业、城建等项目落地开工，促进总投资2220亿元的180个市级重点项目加快建设；争取中央扩内需投资项目130个。落实中央、省扩大消费政策，做好家电、农机、汽车摩托车下乡等工作，社会消费品零售总额1086亿元，增长18.7%。果断采取促进外贸增长10项措施，全力保市场、争订单，外贸出口降幅低于全省11.4个百分点，总量占全省的份额提高。针对企业经营难和融资难，实施增值税转型改革，组织涉企收费专项治理，采取60项税费减免措施，对符合条件的企业缓缴社会保险费和给予社会保险补贴，市本级财政安排扶持企业发展资金3.11亿元，增值税转型让利17.1亿元，减轻企业负担超过20亿元，办理出口退（免）税102.48亿元；推出“金融超市”等12项举措，推动金融机构与1300多家企业达成1500多亿元授信意向，各类金融机构新增贷款681.23亿元，为历史最高水平。及时推出促进房地产回暖优惠政策，大力度推进拆迁安置房建设，加大普通商品房供地力度，推动出让地块加快开工，加强市场监管和消费引导，全市普通商品房销售面积增长59%，拆迁安置房新开工面积342.38万m2、增长31.9%，市区普通商品房用地出让232.45公顷、增长249.9%。全市经济运行好于预期，主要指标增幅高于全省平均水平，GDP、地方一般预算收入、规模以上工业增加值增幅分别高于全省1.6个、6.5个、2.3个百分点。

　　（二）全力抢抓国家战略机遇，新一轮沿海开发全面拉开。抓住长三角一体化发展和江苏沿海开发两大国家战略机遇，迅速组织规划、政策、项目向上对接，修编出台《南通市沿海开发规划》，制定实施《南通沿海开发行动纲要》，全力抓好117项重点工作和100个重大项目，掀起新一轮江海联动开发热潮。

　　以港口建设带动开发。洋口港完成投资43亿元，10万吨级LNG码头和管线桥工程初具规模，30万吨级航道建设研究取得初步成果；吕四港区总体规划获部省批复，东灶港作业区码头建设实质性启动，腰沙-冷家沙海域开发前期研究加快。新通海沙和天生港水道整治开发有序推进。如皋港国家一类口岸正式开放，洋口港、吕四港获批临时开放。全市港口货物吞吐量1.36亿吨。

　　以基础设施支撑开发。崇启大桥完成总投资的38%；江海高速完成总投资的72.3%，新204国道全线通车，328国道和334、225、221省道改扩建加快。沪通铁路进入深化过江方式比选阶段，宁启铁路南通段复线电气化改造、海洋铁路建设快速推进。兴东机场飞行区改扩建通过国家民航局审批。海启高速、通如高速、宁启铁路通启段、连申线航道等项目前期工作取得实质性进展。经多方争取，临海高等级公路、平海干线公路项目列入省计划，取得突破性进展。重大电力设施建设实现新突破，大唐吕四电厂4×60万千瓦超超临界机组获国家发改委核准，我市电力装机容量达到571万千瓦，成为全省重要的电力能源生产基地。

　　以载体打造促进开发。苏通科技产业园正式奠基，一期9.5平方公里基础设施项目开工建设，跨江开发、跨国合作迈出重要一步。在建在谈园区跨江合作项目20个，各县（市）区与上海的园区合作均取得实质性成效。13个省级以上开发区GDP、财政总收入占全市比重分别提高2个、4个百分点。洋口港南通国际产业园正式成立，沿海5个滨海产业园区建设加快。

　　以扩大开放推动开发。积极参加省沿海开发系列专题招商，精心组织港洽会等重大活动和“六百六促”招商竞赛，新增工商登记注册外资55亿美元，注册外资实际到账20.05亿美元。提高利用外资质量，新批1亿美元以上项目8个，新落户世界500强投资项目4个。全面整顿对外劳务市场，加快推动“走出去”，完成外经营业额11.56亿美元，连续14年保持全省领先。继续争创江苏民营经济第一大市，个体工商户总数、私营企业数和注册资本保持全省第二，民营经济增加值、实现税收、完成投资占全市比重分别超过50%、60%、70%，20家企业入围全国民营企业500强。新落户央企投资项目12个，市国有资产经营公司为重点项目建设融资82亿元，其中发行企业债券23亿元。积极推进企业上市，中南建设、罗莱家纺成功上市，新增首发申请过会企业2家。

　　（三）着力推进产业升级，发展方式转变加快。主动利用国际金融危机形成的倒逼机制，把扩内需、保增长与调结构、抓创新、促减排紧密结合，促进经济转型升级。

　　加快发展先进制造业。制定实施船舶海工及重装备等六大产业转型升级规划纲要。大力推进主导产业高端化。建造世界首座第六代超深水海洋钻探储油平台、被欧洲造船界誉为“熔盛型”的40万吨矿砂船等一批船舶海工高端产品。全市规模以上造船完工量567万载重吨、海洋工程及特种船舶完工量253万综合吨，分别增长95.3%、361%。装备制造、电子信息、精细化工加速向产业链上游发展，神马电力复合绝缘子系列产品技术水平国际领先。大力推进新兴产业规模化。新能源、新医药、新材料及节能环保产业产值308亿元，占规模以上工业比重5.1%。风电并网发电装机容量54.4万千瓦，增长34%；光伏电池及组件产量321兆瓦，增长44.9%。大力推进传统产业品牌化。现代纺织、粮油食品、冶金建材等行业优势进一步巩固，南通成为中国家纺指数发布中心；新增省级以上名牌产品70个，驰名、著名商标24件。通过调高调优调强，高新技术产业实现产值1800.73亿元，占规模以上工业比重提高1.9个百分点；年销售收入超30亿元工业企业新增4家，熔盛重工成为我市首家百亿级工业企业。推动建筑业向高附加值、高效益转型，施工产值突破2000亿元，增长26%。

　　加快增强自主创新能力。国家级研发机构实现零的突破，新增省级工程技术研究中心44家、超历年总和，新增院士工作站14个、高新技术企业105家，新增科技孵化器面积105万m2、增加3倍，新增国家级、省级科技孵化器12家，科技创业社区即将建成投入使用。专利授权量9279件、列全省第二，通过国家知识产权试点市验收。我市连续5次被评为全国科技进步先进市。制定实施“江海英才”计划。市长质量奖评选正式启动。

　　加快推动集约节约发展、提升节能减排水平。强化土地集约利用，省级以上开发区投资强度254万元/亩，增长11.4%；“万顷良田建设工程”试点全面启动，连续18年保持耕地占补平衡。促进产业集中布局，10个百亿级产业板块、50个重点工业集中区销售收入，占规模以上工业比重分别提高2个、2.5个百分点。实施329个节能减排项目。加大重点行业、重点区域环境整治力度，七大化工园区企业集中度提高10.8个百分点；市区姚港化工区三类企业提前1年完成搬迁改造，西城区集中供热工程完成。推进生态市创建，“城市环境综合整治定量考核”连续6年列全省第一，新增全国环境优美乡镇18个、省级生态村17个。

　　加快促进服务业发展。制定实施《加快市区服务业发展三年行动计划》，市区服务业增加值占GDP比重提高到39.4%。全市服务业增加值1028.84亿元，增长14.3%，连续2年高于GDP增幅。全力抓好72个市级服务业重点项目，规模以上服务业投资442亿元，增长34.9%。新增省级服务业集聚区2家。现代物流业增加值增长15%。新引进金融机构3家。研究出台《服务外包发展战略和产业规划》，新引进服务外包企业超过100家，服务外包营业额增长116.8%，我市成为全省江北首个省级国际服务外包示范城市。市区一批高星级酒店项目加快建设。狼山、濠河创5A级景区等一批旅游项目快速推进，“江与海的交响”等5条精品线路入选世博旅游推荐线路，实现旅游总收入188亿元，增长24.7%。

　　（四）大力加强“三农”工作，新农村建设扎实推进。

　　大力调整农业结构，推动现代高效农业上规模、上水平。粮食连续六年丰收、综合单产创历史新高；规模以上“三资”开发农业投资102.85亿元，列全省第一；新增高效农业面积29.1万亩，其中设施农业11.96万亩；新增高效渔业面积12万亩。农业综合开发投资历年最多，农机装备水平进一步提高。

　　深入推进农村综合改革，增强农村发展活力。加强农业服务体系建设，着力构建四级农业技术推广网络。提高农村经济组织化程度，新增农村三大合作组织1040家、农村土地流转面积52万亩。强化农村金融服务，小额贷款公司实现县（市）区全覆盖。进一步加强农村基层组织建设和经营管理，村级公共服务中心功能不断完善，村级债务下降6.7%。供销服务网络、粮食流通体系进一步健全。

　　加强农村基础设施和环境建设。新建农村公路900公里，新建、改造农村公路桥梁和农用桥梁984座。县（市）新建污水处理厂25家，新增污水日处理能力16万吨，乡镇污水处理设施覆盖率突破50%。扎实推进新一轮农村六件实事，农村河道整治提前一年完成省下达任务，农村改厕超额完成年度任务，农村生活垃圾“户集、村运、镇处理”机制进一步健全。新增造林面积25万亩，高标准建设“绿色通道”249公里，森林覆盖率提高到20%。积极稳妥推进农民集居区建设，新增竣工面积580万m2、入住农户2.9万户。

　　（五）加大城市建设力度，中心城市辐射力增强。

　　强化规划龙头作用。以通州撤市设区为契机，迅速修编形成新一轮城市总体规划，编制完成市区分区规划、城市近期建设规划等4类22项规划，开展县（市）城总体规划修编和省级重点中心镇规划编制，积极构建大城市空间格局。

　　突出提升中心城市功能。组织实施106项城建项目，完成投资超100亿元。加快推进以通沪大道高架等快速通道为重点的68项路桥工程，市区新增道路面积189万㎡。新城区建设提速，中央商务区和能达商务区分别完成投资23.6亿元、2.1亿元；老城区改造步伐加快，十字街综合改造、老小区整治等工程陆续实施；北翼新城和通州区城东新区建设有序推进。加强城市环境和公用设施建设，制定并启动市区“清水工程”三年行动计划，完成12项河道整治工程；市区更新公交车180辆，新增绿地300公顷，新增污水日处理能力12.5万吨。

　　2009年1月20日我市荣获全国文明城市称号。我们以此为动力，深入推进精神文明创建工作，不断提高城市管理水平。积极开展“迎国庆、讲文明、树新风”活动，城市公共文明指数测评列全省地级市首位。历史文化名城保护规划获省政府批准。着力构建“大城管”格局和城市管理长效机制，市级数字化城管平台建成并投入使用。

　　统筹推进中心城市与县（市）城、重点中心镇、小集镇建设。全市城市化率52.7%，提高2.4个百分点。支持县（市）城完善城市功能、加速人口集聚，各县（市）城完成城建投资总和超过100亿元。大力培育重点中心镇，一批临港新镇、工贸强镇、旅游名镇特色更加明显，对农村发展的示范带动力进一步增强。

　　（六）重视改善民生工作，社会公共服务和管理进一步加强。把改善民生作为“保增长、达小康”的落脚点，持续加大民生投入，全市财政在就业、社会保障和教育、医疗、文化事业等方面的投入139.74亿元，增长24.1%，比地方一般预算支出增幅高3.8个百分点。12件26项政府为民办实事项目全面完成（见附件一）。在市人大代表“加快实现城乡居民普遍饮用长江水”1号议案的推动下，新增区域供水受益乡镇24个、近100万人，第二批70万农村居民饮水安全问题得到解决。

　　大力促进就业。以开展充分就业社区创建和实施促进就业五项工程为抓手，通过服务企业、鼓励创业、发展经济创造就业岗位，通过落实优惠扶持措施稳定就业岗位，通过政府购买服务开发就业岗位，新增城镇就业8.2万人，帮助创业近2万人，促进城镇失业人员再就业3.8万人，城镇登记失业率连续11年低于全国、全省水平；全市城镇95%以上社区建成充分就业社区；农村劳动力转移新增4.6万人，总量继续保持全省领先。

　　积极完善“广覆盖、保基本、多层次、可持续”的社会保障体系。城镇职工基本养老、基本医疗、生育、工伤、失业保险和城镇居民医疗保险覆盖率均超过97.5%；新型农村社会养老保险参保人数113万人，参保率97.6%，居全省前列；新型农村合作医疗参合率99.2%，市区新型农村合作医疗和城镇居民医疗保险实现并轨，在通大学生全部纳入城镇居民医疗保险；为市区城镇老年居民发放养老补贴。再次提高企业退休人员养老金和城乡低保标准。深入推进脱贫攻坚工程，农村贫困人口减少14.25万人。

　　加快发展社会事业。全面推进区域教育现代化，8个县（市）区接受省级评估。编制完成教育中长期改革发展规划。义务教育阶段绩效工资兑现到位，优质普通高中建成率100%。职业教育服务地方发展能力得到增强。南通大学成为交通运输部和省政府共建大学。提高公共卫生服务水平，市区实现基本药物零差价销售，新一轮农民健康工程启动；甲型H1N1流感等重大疫情防控有力有效。人口自然增长率-2.7‰。推进城乡公共文化服务体系建设，乡镇综合文化站和村文化室、“农家书屋”全覆盖，成功承办第四届尼泊尔中国节。文化精品创作在省“五个一”工程七个门类中均有获奖。县（市）有线电视数字化整体转换率接近30%。全民健身和南通体育日系列活动深入开展，我市在全省江北率先建成体育强市。大力推进社会事业重点项目建设，市第六人民医院基本建成，妇幼保健院迁建完成总体设计；报业新闻传媒中心、图书馆新馆奠基，市民服务中心主体封顶。老龄事业加快发展，职工权益维护、妇女、儿童、青少年、残疾人、关心下一代、见义勇为、法律援助、红十字和慈善等工作进一步加强。对口援建四川地震灾区一年完成三年投资任务的70%。

　　深入推进平安南通建设。主动化解社会不稳定因素，大调解机制、大防控体系不断完善，矛盾纠纷调处成功率上升，万人发案率下降，公众安全感98.8%，平安南通成为百姓认可的社会品牌、经济发展的重要优势。2009年5月18日我市获得全国社会治安综合治理“长安杯”。法治城市创建全面启动。安全生产事故起数和死亡人数连续8年双下降。社会应急管理体系进一步健全。食品药品监管扎实有效。居民消费价格指数下降1.3%。国防教育、民兵预备役、优抚安置、人防（民防）、国家安全等工作取得新成绩，6个县（市）区荣获全省双拥模范城称号。民族、宗教、外事、侨务、港澳事务、对台事务、地方志、统计、气象、防震减灾、无线电管理、档案、保密、新闻出版、哲学社会科学、机关事务管理等工作继续加强。

　　各位代表！过去一年，我们认真开展深入学习实践科学发展观活动，积极弘扬新时期“南通精神”，努力提高工作创造力、行政执行力和政府公信力。坚持把上级要求和南通实际紧密结合，以创新的思路和办法应对危机、克服困难。针对性地加强调查研究，30多项调研成果转化为化解难题的对策措施。创新重大项目办公会和重点企业会办会等推进机制，完善“12345”政府服务热线、市长信箱、市长接待日、群众来信来访、网上与群众直接交流的民生诉求服务机制，努力提高服务企业、服务群众效能。加大督查力度，127项重点工作全面完成。加强政府法制建设，废止、修改不适应发展要求的政策性文件845个。深入推进政务公开，实施行政权力网上公开透明运行。自觉接受市人大及其常委会的法律监督、工作监督和市政协的民主监督，认真执行市人大及其常委会决议，支持市政协参政议政。对市人大代表1号议案和“大力培育支柱产业，提升区域经济竞争力”2号议案，市政协“优化投资结构，增加有效投入”、“推进新型城镇化”建议案，建立市长、副市长牵头承办责任制，加大投入和推进力度，确保有关目标任务落到实处。高度重视、圆满完成人大代表建议、政协委员提案办理任务。全面推行政府投资项目代建制，深入开展工程建设领域突出问题专项治理，强化用人责任、经济责任、编制责任联合审计。推动节约型机关建设，市级机关公用经费压缩11%。

　　各位代表！我们清醒地看到，我市经济社会发展仍然面临严峻挑战。经济结构中的深层次矛盾还比较多，高端产品、新兴产业比重还不高，相当数量的企业创新能力还不强，支撑发展的大企业（集团）还不多；城市化进程还需加快；城乡居民持续增收难度较大，部分群众生活比较困难，抑制普通商品房价格过快上涨还需作艰苦努力；随着经济环境变化和社会转型，社会管理面临不少新情况；政府职能还需加快转变，少数部门和工作人员为企业和群众服务的主动性、创造性还有待增强，极少数人甚至违法违纪。对此，我们一定高度重视，认真解决。

　　各位代表！过去一年，是经受重大考验、取得重大收获的一年。我们不仅取得了超出预期的发展成果，向新中国成立60周年献上了一份厚礼，而且积累了在复杂环境中推动经济社会又好又快发展的宝贵经验，为今后工作奠定了坚实基础。这是在市委的正确领导下，市人大、市政协的监督支持下，全市人民风雨同舟、共克时艰的结果。在此，我谨代表市人民政府，向广大干部群众，向人大代表、政协委员和离退休老同志，向市各民主党派、工商联、各人民团体和各界人士，向人民解放军、武警驻通部队官兵、公安干警和部省驻通单位，向所有关心和支持南通发展的海内外朋友，致以崇高的敬意和衷心的感谢！

二、2010年目标任务

　　在全省江北率先以市为单位总体达到全面小康标准，标志着我市提前实现了“十一五”规划确定的“全面达小康、建设新南通”的预期目标。站在新的历史起点，市委十届六次全会按照《江苏沿海地区发展规划》和省委、省政府对南通“两个率先”的要求，提出用9年左右时间，建设一个“经济更加强盛、科教更加发达、社会更加文明、文化更加繁荣、生态更加优良、人民更加幸福的基本现代化新南通”。今年是开启基本现代化建设新征程的第一年，也是实施“十一五”规划的最后一年。这一年，经济环境中不确定、不可预料因素仍然较多，我们将保持清醒头脑，做好应对各种困难和挑战的准备。这一年，长三角一体化发展和江苏沿海开发两大国家战略全面实施，上海世博会正式举办，迈入“桥港新时代”的南通具有更多发展有利条件。我们将倍加珍惜利用好历史机遇，放大好比较优势，延续好发展态势，让全面小康成果更多惠及老百姓。

　　今年政府工作的总体要求是：全面贯彻党的十七大和十七届三中、四中全会精神，深入贯彻落实科学发展观，按照省委、省政府和市委一系列决策部署，抢抓长三角一体化发展和江苏沿海开发两大国家战略机遇，在提高经济增长质量和效益、加快经济转型升级、统筹城乡区域发展、深化改革扩大开放、改善民生维护稳定上下更大功夫，加快推动经济发展方式转变和由全面小康向基本现代化迈进，全面完成“十一五”各项发展目标，奋力开启基本现代化建设新征程，努力开创科学发展新局面。

　　今年全市经济社会发展的主要预期目标是：GDP突破3200亿元，增长13%左右；财政总收入突破560亿元，其中地方一般预算收入增长15%；全社会固定资产投资增长20%，外贸出口总额增长10%，社会消费品零售总额增长17%；城镇居民人均可支配收入、农民人均纯收入均增长10%，居民消费价格指数涨幅3%以内，城镇登记失业率4%以内；全社会研发投入占GDP比重1.8%左右，高新技术产业产值占规模以上工业比重30%以上；万元GDP综合能耗下降4%左右，主要污染物COD、SO2排放总量下降幅度完成省下达任务。与往年比，今年首次把全社会研发投入占GDP比重、高新技术产业产值占规模以上工业比重作为预期目标，主要是强化科学发展导向，把着力点更好地放到依靠科技进步，转变发展方式，提升发展质量上来。

　　按照上述要求和目标，我们将更加突出转变发展方式，奋力打造经济竞争新优势；更大力度推进沿海开发，拓展经济发展新空间；更高水平扩大开放，增强发展新动力；更好统筹发展，加快城市化和城乡一体化新进程；更加注重改善民生，加强社会建设和管理，努力形成社会和谐稳定新局面。

　　（一）更加突出转变发展方式，奋力打造经济竞争新优势。把转变发展方式作为今年工作的重点和决定我市未来经济发展的关键，加快向创新型经济、绿色低碳经济转型升级。

　　狠抓支柱产业向高端升级。实施船舶海工及重装备、能源新能源及其装备制造两大产业“双倍增”计划。推动船舶海工产业向“高技术、高附加值、高配套、较大经济规模”方向发展，重点扶持发展深海钻井平台、LNG船、豪华游艇及超大型船舶等高端产品，拉长船舶海工配套产业链，加快建设国际先进、国内一流的船舶海工及重装备制造基地；扶持发展高端输变电设备、能源新能源制造及装备，加快建设长三角重要的能源及装备制造基地，力争2-3年内船舶和海洋工程综合吨位产量、新能源和节能环保技术装备及配套产值实现倍增目标。推动电子信息、石化及新材料两大产业“双突破”。着力突破终端产品占比相对偏低和具有国际先进水平高端产品相对偏少的状况，加快建设国内一流的电子信息产业基地和全国重要的新兴石油化工及精细化工产业基地。促进纺织、轻工两大产业“双提升”。支持企业加强技改，引导产业链向研发设计和营销服务两端延伸，提升产品附加值，提升产业品牌化水平。积极推进建筑业转型升级，加快产业链向关联度高、附加值高的上下游延伸。

　　狠抓新兴产业拓展规模。研究制定加快我市战略性新兴产业发展意见，重点支持新能源、新医药、新材料及节能环保产业，大力培育智能装备、海洋技术、生物工程产业，力争规模以上新兴产业产值突破450亿元、占规模以上工业比重提高1个百分点以上。加大风电产业发展规划统筹力度，着力打造“海上风电三峡”，力争风电并网发电装机容量80万千瓦以上。重点发展高转换效率光伏电池及组件，抓好一批太阳能发电示范项目，力争2-3年光伏电池及组件产能1000兆瓦以上。集中力量主攻生物医药、新型合成材料等具有竞争优势的领域，形成一批新兴特色产业集群。对战略性新兴产业实行政策优先扶持、资金优先安排、技术人才优先引进，引导优质要素向新兴产业集聚。

　　大力提高现代服务业在三次产业中的比重。现代服务业发展的重点在市区，在县（市）城、中心镇。认真落实加快市区服务业发展三年行动计划，力争市区服务业增加值占GDP比重突破40%，全市服务业增加值占GDP比重超过36%。突出提高物流附加值，加快发展第三方、第四方物流，积极培育出口加工区保税物流，力争物流总额增长20%以上，港口货物吞吐量突破1.5亿吨。高度重视发展旅游业，抓住上海举办世博会的契机，把旅游业与会展、商务服务等结合起来，加快新建一批4A、5A级景区，加大旅游资源商品化开发、高星级酒店建设力度，吸引更多海外、市外游客，着力打造独具特色的江海旅游门户城市。以建设文化强市为目标，大力实施文化建设规划纲要，推进文化产业园区和基地建设，积极发展广播影视、创意动漫、出版发行、演艺娱乐、工艺美术、民俗博览等文化产业。推进实施服务外包发展规划，重点发展研发、软件、呼叫等产业，力争服务外包营业额增长30%以上。改造提升批发、零售、住宿、餐饮等传统服务业，促进城乡消费升级。加快培育壮大21个市级以上服务业集聚区，抓好110个市级服务业重点建设项目和50个重大前期项目。

　　大力提升自主创新能力。着力培育自主知识产权和自主品牌，做大做强科技型企业，确保高新技术产业产值突破2000亿元。建设一批高层次创新平台和科技园区，确保科技孵化器面积达到200万m2。推动企业研发平台建设，引导支持企业加入产业技术创新联盟和技术标准联盟，完善以产业为纽带的产学研合作机制。大力实施“江海英才”计划，加强高层次人才队伍建设，力争引进高层次创新创业人才150名、新建“院士工作站”6个。

　　大力强化节能减排、环境保护工作。加快淘汰落后产能，从严把好项目节能评估和环保准入关。确保新增污水日处理能力20万吨，全市生活污水集中处理率和农村生活垃圾无害化处理率均提高10个百分点。严格化工集中区污染排放管理，深入开展小化工、小印染企业污染整治，对不符合条件的一律关停，对违法违规排放的依法严惩。启动实施城市水系规划，加快推进市区“清水工程”三年行动计划。积极增加森林碳汇，力争森林覆盖率提高1个百分点。加快生态市创建步伐，力争基本达到国家生态市考核标准，确保3-4个县（市）基本达到国家生态县考核标准。

　　（二）更大力度推进沿海开发，拓展经济发展新空间。按照“当好全省沿海开发主力军，打造江苏新的增长极”的定位，组织好沿海开发大会战，切实落实沿海开发行动纲要。

　　建设大港口。启动洋口港30万吨级航道规划建设工作，加快腰沙-冷家沙港口开发前期工作。开工建设洋口港10万吨级航道和推进吕四港挖入式港池及进港航道上延工程，抓好洋口港10万吨级LNG码头、10万吨级液体化工码头和吕四港5万吨级散货码头、东灶港作业区2个2万吨级通用码头建设，加快推进新通海沙岸线整治工程和狼山港三期集装箱泊位、如皋港公用码头等11项工程建设，确保5万吨级以上码头新增5座，总数突破40座。

　　培育大产业。大力实施六个“千亿级”产业板块培育计划，制定促进海洋经济发展意见。以大项目带动大产业，重点推进3类各80个市级重点项目。突出抓好总投资超百亿元的王子制纸、恒力PTA、大唐吕四电厂和总投资超50亿元的LNG接收站、熔盛海工等重点项目建设，力争醋纤五期等项目早日获批开工。跟踪争取电动汽车、金鹰差别化纤维、宝钢工业园等10个百亿级产业项目。做强重点载体集聚大产业。按照“江海生态城、国际创业园”的定位，全力推进苏通科技产业园建设，力争基础设施一期工程基本完成、二期工程全面启动。跟踪抓好20个园区跨江合作项目。实施园区跨越三年行动计划，强化产业布局规划引导、特色园区培育，促进国家级开发区“三次创业”和12个省级开发区“二次创业”、争先晋位，推动5个滨海产业园区提升功能、错位发展，开工建设洋口港南通国际产业园，做大做强重点工业集中区，增强对重大项目的吸引力和承载力。

　　构筑大通道。全力争取沪通铁路尽快开工，宁启铁路南通段复线电气化改造和海洋铁路建设分别完成投资8亿元、6亿元。确保兴东机场飞行区改扩建完成总工程量的60%。继续做好崇启大桥建设服务工作；确保江海高速、328国道建成通车，221、225省道改扩建完成路基工程，336省道改扩建工程全面开工；实施总投资60亿元临海高等级公路和平海干线公路东段改扩建工程。启动连申线航道南通段整治工程。加快崇海大桥、海启高速、通如高速、宁启铁路通启段、南通客运枢纽东站等项目前期工作。

　　滩涂大开发。抓紧编制《南通沿海滩涂匡围开发规划》，实施洋口港太阳岛三期等5个滩涂围垦项目，推进金牛岛、腰沙-冷家沙垦前工作。坚持生态开发、集约节约开发，积极探索市级统筹、市县联动、共建共享新机制。设立市级沿海开发引导基金，巩固多元化开发格局。今年力争围垦滩涂20万亩，为沿海产业发展提供用地保证。

　　（三）更高水平扩大开放，增强发展新动力。充分利用两种资源、两个市场，充分激发市场主体活力，确保注册外资实际到账25亿美元，引进市外民资400亿元。

　　提升开放型经济国际竞争力。一是促进对外贸易转方式、扩份额。提高自主知识产权、自主品牌产品出口比重，培育一批出口超亿美元企业、超10亿美元特色产业和国家级、省级出口基地。认真落实出口退税、信用保险、信贷等政策措施，继续优化海关、质检等服务，扩大外贸出口在全省的份额。二是促进利用外资调结构、提质量。注重产业链招商，围绕优势特色产业，招引一批产业关联度高的旗舰型、龙头型项目，催生一批创新型项目，培育一批龙头、配套企业。注重放大资源性优势招商，精心组织港洽会、世界船舶高层峰会等专题招商活动，引导外资更多投向沿海开发领域。注重营造比较优势和环境招商，以生产要素成本优势、国际化配套服务和便捷舒适人居环境，吸引一批跨国金融、贸易、投资等机构落户。创新利用外资方式，鼓励引进私募、创投基金，鼓励境外上市，鼓励外资企业增资扩股，培育一批根植型企业；推动引资与引智力、引业态结合，大力招引各类研发中心、采购中心等地区性总部落户。三是促进“走出去”上水平、拓空间。加快完善境外投资促进体系，努力推动由劳务、产品输出为主到产业、技术、资本、品牌等集成化、规模化输出的提升。强化“走出去”的引导和服务，鼓励过剩产能有序向外转移，鼓励有条件企业收购境外知名品牌、营销网络和研发机构。

　　放大民营经济领先优势。以争创江苏民营经济第一大市为抓手，提升民营经济总量和质态优势。一是大力弘扬“三创”精神，充分激发群众创业激情。积极探索放开民资准入领域，降低准入门槛。加大财税、担保等政策支持，完善“金融超市”等中小企业融资机制，为创业提供环境和平台。二是大力提升民营经济板块竞争力。进一步做强家纺、电动工具等10个百亿级优势特色板块，重点扶持一批发展基础好的专业大市场，促进集聚化发展。培育一镇一品、一村一品，支持做强区域特色产业板块。三是大力推动争创“三名”。支持民营企业加强与国内外大企业（集团）合作，培育一批领军型企业，力争年销售收入超亿元民营工业企业新增160家，总数达到700家；培育更多名牌产品和优秀民营企业家，推动民营经济提质增效。

　　加大重点领域改革力度。推进市县政府机构改革，深化乡镇机构改革。实施新一轮市区财政体制。加快组建南通农村商业银行，推动农村信用社体制改革；全力争取在通设立沿海开发银行，新引进银行机构2-3家，实现外资银行落户零的突破。加大企业上市推动力度，力争上市企业新增5家，总数达到20家，形成具有相当规模的南通上市板块。支持有条件的企业发行债券。确保国有资产保值增值。稳妥推进文化体制改革。认真实施事业单位全员聘用和绩效工资改革。

　　（四）更好统筹发展，加快城市化和城乡一体化新进程。

　　加大中心城市建设力度，进一步发挥对城乡统筹发展的带动作用。按照新一轮城市总体规划“建设我国东部沿海江海交汇的现代化国际港口城市、上海北翼的经济中心和门户城市、国内一流的宜居创业城市、历史与现代交相辉映的文化名城”的定位，不失时机掀起新一轮城市（镇）化建设热潮，力争城市化率提高2个百分点。着力增强中心城市集聚辐射功能，提高市区经济总量在全市中的比重。以推进110项城建项目为抓手，加大老城区商业中心改造力度，完成十字街综合改造一期工程，显著提升南大街核心商圈功能和形象；加快新城区商务中心建设，高标准打造标志性空间和载体，增强对各类要素的吸引力和集聚力；完善通州区、港闸区、开发区服务配套功能，落实扶持通州区和其他三区加快发展的政策，推动四个区联动发展。编制新一轮城市道路交通规划，积极推动“干道快捷化、道路网络化、交通立体化、管理手段设施化”，组织市区快速路网建设大会战，完成通沪大道高架工程，开工建设通宁大道、外环北路快速化改造等快速路工程，实现主城区机动车辆在20分钟内能上高速；推进70条次干道、支路建设；强化交通组织管理，完善交通分流、公共停车等设施；新辟、优化一批公交线路，规范发展出租车行业，提高公交出行分担率。加强宜居环境建设，加快推进一批“城中村”和危旧房改造、老小区整治工程，建设环保公园等一批绿化景观工程；加快实施寺街·名人文化区、南通·1895等一批历史文化保护建设工程，做好国家历史文化名城保护工作。完善数字化城管平台，加强市容环境、小区物业管理，提升城市精细化管理水平。

　　加强县（市）城和重点中心镇建设，提升对城乡统筹发展的承载能力。一是加强县（市）城和中心镇基础设施建设，提高城镇的发展质量和水平，进一步增强公共服务、产业承载和吸纳人口就业能力。二是提高城镇集约发展水平。完善县（市）城总体规划和重点中心镇建设规划，进一步优化城镇空间布局。坚持最严格的耕地保护和节约用地制度，促进工业向园区集中、人口向城镇集中、居住向社区集中，积极探索解决“三集中”遇到的新情况新问题，努力提高城镇土地利用率。注意发挥好小集镇基础设施较好、规模相对成型等优势，示范带动农民集居点加快建设。三是推进户籍制度改革，研究制定加快城市（镇）化的配套政策，逐步解决农村人口在城镇的就业和生活问题，加快农村劳动力向非农产业和城镇转移。在有条件地区积极探索开展土地承包经营权置换城镇社保以及宅基地、住房置换城镇住房改革试点。

　　扎实做好“三农”工作，夯实城乡统筹发展基础。继续落实强农惠农政策，深入推进“民富、村美、风气好”新农村建设。一是大力发展现代高效农业。以项目农业为抓手，打造高效农业规模优势，力争形成10亿元以上规模产业不低于15个，确保新增高效农业面积25万亩，规划建设万亩以上园区10个、千亩以上设施农业基地20个。加大农田基础设施建设和农业综合开发力度，积极发展市区都市生态型现代农业。二是加强农村基础设施建设。继续办好新一轮农村六件实事。新建农村公路1200公里，新建、改造农村公路桥梁和农用桥梁600座以上。继续加快推进区域供水和农村饮水安全工程，新增日供水能力40万吨，新增区域供水受益人口80万人，完成第三批70万农村居民饮水安全建设任务。深化农村环境综合治理，继续抓好农村河道疏浚，全面推行农村河道、道路、绿化、垃圾清运“四位一体”长效管理机制。三是多渠道增加农民收入。把促进农民就业创业作为最大的农民致富工程，加快推进劳动保障服务平台和促进就业创业政策体系向农村延伸，村级劳动保障服务平台实现全覆盖，确保50%的行政村建成“充分就业村”。大力发展村级集体经济，拓展农民增收渠道。深入实施脱贫攻坚工程，继续抓好扶贫和老区开发，基本消除绝对贫困现象。四是深化农村各项改革。坚持农村基本经营制度，推进“万顷良田建设工程”，促进农业适度规模经营。发展农村三大合作组织，力争农民专业合作组织入社率提高4个百分点、社区股份合作制改革面达到80%、新增土地股份合作社100家。创新农村金融保险服务，积极发展农村小额贷款公司，开展农民资金互助合作试点，扩大农业政策性保险试点。

　　（五）更加注重改善民生，加强社会建设和管理，努力形成社会和谐稳定新局面。进一步推动公共资源配置向就业、社会保障和教育、医疗等民生领域倾斜，确保地方一般预算支出的50%以上用于民生。坚持为民办好10件27项实事（见附件二）。

　　着力强化就业和社会保障工作。把就业作为民生工作的头等大事，完善财税、信贷、社保补助等扶持政策，支持企业增加就业岗位，创造条件帮助城镇劳动者自谋职业、自主创业，开发公益岗位帮助城镇就业困难人员就业，确保新增城镇就业岗位7万个、城镇零就业家庭动态为零。大力创造面向大学生的智力密集型就业机会，引导高校毕业生到基层就业、到中小企业就业和自主创业，确保高校毕业生年终总就业率不低于90%。继续扩大社会保险覆盖面，积极推进灵活就业人员、农民工等参加城镇职工基本养老保险，将城镇居民纳入生育保险，进一步完善新型农村社会养老保险制度。提升社会保障水平，提高企业退休人员养老金，对城镇老年居民发放养老补贴。关心关注弱势群体，完善城乡低保、农村五保户、被征地农民生活保障标准自然增长机制。

　　着力解决群众关注的住房问题。一是加大保障力度。继续高效率、高质量推进拆迁安置房建设，确保跟上城市建设和拆迁户安置的需要，全市拆迁安置房新开工410万m2以上、竣工交付300万m2以上。增加廉租住房实物配租和经济适用房供应，继续保证符合住房保障政策的申请对象“应保尽保”。二是加大要素供给力度。把土地作为调控房地产市场的重要手段，适时增加住宅用地供应，加快中低价位、中小套型普通商品房建设，增加普通商品房有效供给。三是加大监管引导力度。充分发挥金融、税收等政策措施对房地产市场调节的杠杆作用，进一步依法规范房地产市场秩序，保持房地产市场平稳健康发展。

　　着力解决群众关注的看病、养老问题。进一步优化城乡医疗资源布局；加快实施市区新城区医院和市妇幼保健院迁建等项目，支持优质医疗资源向社区延伸，提高社区卫生服务水平，促进公共卫生服务均等化。抓好医药卫生体制改革，开展公立医院改革试点，推进实施国家基本药物制度，降低群众看病负担。确保新型农村合作医疗人均筹资标准提高到160元，其中政府补助不低于120元，住院费用实际补偿比例45%以上。建立城镇职工和居民医保门诊统筹制度，城镇职工医保和居民医保制度规定范围内医药费用报销比例分别达到80%和60%。加快发展老龄事业和老年服务产业，加强养老服务体系、设施建设，实现市区居家养老服务站全覆盖。

　　大力发展社会事业。启动实施教育中长期改革发展规划，提升区域教育现代化水平。推进中小学布局调整优化，促进基础教育均衡协调发展。完成中小学校舍安全改造150万m2。办好学前教育和特殊教育。制定新一轮职业教育发展规划，加强示范专业和实训基地建设，促进职业教育优化发展。积极支持南通大学建成国内有影响的综合性大学，加强各所高校重点学科、重点实验室建设。加大扶困助学力度，确保家庭经济困难学生就学。强化城乡公共文化服务体系建设，加快图书馆新馆、凤凰书城、工青妇文化活动中心等一批文化设施建设，探索建立乡镇综合文化站和村文化室、“农家书屋”长效管理机制。基本完成县（市）有线电视数字化整体转换，推进农村乡镇广播电视站规范化建设。巩固提升全国文明城市创建成果，广泛开展群众性精神文明创建活动。加强社会主义核心价值体系和未成年人思想道德建设，弘扬新时期“南通精神”，光大精神文明“南通现象”。贯彻落实国务院《全民健身条例》，全面备战省十七运。实施计划生育优质服务，做好省人口协调发展先进县（市）区创建工作。开展第六次人口普查。积极发展妇女、儿童、青少年、残疾人、见义勇为、红十字和慈善等事业。推进民族、宗教、外事、侨务、港澳事务、对台事务、气象、防震减灾等事业协调发展。扎实做好地方志、哲学社会科学、关心下一代、无线电管理、档案、保密、机关事务管理等工作。完成对口援建四川地震灾区任务。

　　全力维护社会稳定。更加注重社会矛盾化解，深化大调解机制建设，切实加强信访工作，建立社会稳定风险评估机制，从源头上减少和预防社会矛盾发生。更加注重基层基础建设，发挥乡镇街道政法综治中心功能，实现问题联处、矛盾联调、治安联防、平安联创。更加注重社会组织建设和管理，建立完善以专业社工、心理矫治为主抓手的社会管理服务新平台。更加注重流动人口社会化服务和管理，提高外来人员管理服务中心综合服务水平。更加注重加强长安南通建设，提升大防控体系建设和公正廉洁执法水平，依法惩治各种违法犯罪行为。完善应急管理机制，提高突发事件预警处置能力。强化安全生产监管。认真落实食品安全监管责任，深入开展药品安全整治。继续做好甲型H1N1流感等重大传染病防控工作。加强关系民生的重要商品价格监管和市场调控。推进法治南通、诚信南通建设，完成“五五”普法任务，完善社会信用体系。支持国防和部队建设，开展全国双拥模范城“五连冠”创建活动，做好国防教育、征兵、民兵预备役、人防（民防）、国家安全、反邪教等工作。

　　各位代表！做好“十二五”规划的编制工作，是今年的一项重要任务。我们将在全面完成“十一五”各项目标的同时，充分发扬民主，集中各方智慧，认真谋划好下一个五年经济社会发展的思路、目标和举措，全力推进新一轮又好又快发展。

　　各位代表！新的形势和任务对政府工作提出了更高要求。我们将进一步加强自身建设，勤勉尽责，奋发进取，为巩固全面小康成果，推进基本现代化建设不懈努力。以创新的举措服务发展，提高工作创造力，建设服务型政府。坚持执政为民，完善民生诉求服务机制，有效解决涉及群众利益的突出问题。更好服务企业，服务项目推进。推动行政审批提速增效，加强电子政务建设，全面实施行政权力网上公开透明运行；完善项目并联审批、限时办结等制度。以务实的作风抓好落实，提高行政执行力，建设责任政府。坚持求真务实，加强调查研究，说实话、出实招、办实事、求实效。健全抓落实的工作机制，加强重点工作督查考核，严格责任追究，强化各级政府的执行力，确保各项工作落到实处。以良好的形象取信于民，提高政府公信力，建设法治政府、廉洁政府。着力提高依法行政水平，进一步规范行政行为。扩大政府投资项目、公共政策、公共服务等信息公开，坚持重大事项集体决策、专家咨询、社会公示和听证等制度。自觉接受人大及其常委会的法律监督和工作监督，主动接受政协的民主监督，高度重视办好人大代表议案、政协建议案和人大代表建议、政协委员提案。广泛听取各民主党派、工商联、无党派人士意见，充分发挥工会、共青团、妇联等人民团体和行业协会、商会的积极作用。坚持艰苦奋斗，廉洁从政，从严治政，认真贯彻落实中央《党员领导干部廉洁从政若干准则》和《关于进一步从严管理干部的意见》，主动接受各方面监督，强化制度约束，坚决查处各类违法违纪案件，保持公务员队伍为民务实清廉的良好形象。

　　各位代表！南通新的发展征程已经开启，我们肩负的使命光荣而重大。让我们紧密团结在以胡锦涛同志为总书记的党中央周围，在省委、省政府和市委的坚强领导下，团结拼搏，锐意进取，为全面实现“十一五”目标，加快向基本现代化迈进而努力奋斗！
